# Early implanon discontinuation and associated factors among women ever used implanon in Mettu district, Oromia regional state, southwest Ethiopia, 2021

**DOI:** 10.1186/s12978-021-01222-8

**Published:** 2021-08-28

**Authors:** Hana Tesfaye, Ebissa Negara, Kenbon Bayisa

**Affiliations:** 1 Midwifery Department, College of Public Health and Medical Science, Mettu University, Metu, Ethiopia; 2 Public Health Department, College of Public Health and Medical Science, Mettu University, Metu, Ethiopia

**Keywords:** Implanon, Contraceptive, Early implanon discontinuation, Mettu district

## Abstract

**Background:**

Implanon is an effective form of long-acting reversible contraceptive used to prevent conception with a clinical failure rate of less than one per 100 users. However, in sub-Saharan countries the utilization of implanon was very low. Regardless of low utilization; its early discontinuation is very common in most developing countries including Ethiopia.

**Objectives:**

To assess the prevalence of early implanon discontinuation and associated factors among women ever used implanon in Mettu district.

**Methods:**

A community based cross-sectional study design was conducted from October 11 to December 4, 2020 G C. A total of 430 women were included in the study by systematic random sampling technique. Data were entered into epi data version 3.1 and analyzed by SPSS version 25.0. Descriptive analysis was computed to describe descriptive results. Logistics regression was computed to see the relative effect of factors on the outcome variable. Adjusted odds ratio was calculated with 95% confidence intervals to show strength of association and p-value < 0.05 was used to declare statistical significance. The finding of the study was presented using narrations, tables and chart.

**Result:**

The total proportion of early implanon discontinuation among 430 mothers was 19.3%. Women who did not counseled about the presence of alternatives methods [AOR = 2.28: 95% CI (1.22–4.26)], women who experienced dizziness after insertion of implanon [AOR = 1.90: 95% CI (1.06–3.43)] and being having menstrual disturbance after insertion of implanon [AOR = 2.17: 95% CI (1.16–4.08)] were significantly associated with early implanon discontinuation. Women who were counseled about the advantage of implanon [AOR: 0.49: 95% CI (0.28–0.87)] were protective from early implanon discontinuation.

**Conclusion and recommendation:**

Early implanon discontinuation among mothers was found to be high. Hence, effective counseling on advantages and side effects of implanon and proper management of the side effects should be made to increase implanon retention.

## Plain language summary

Worldwide, a large number of women become exposed to the risk of conception after contraceptive discontinuation. Besides its low utilization, the discontinuation rate of implanon in developing country was high including Ethiopia. There is no study conducted in south western Ethiopia which shows the magnitude and associated factors of early implanon discontinuation. Therefore, this article provides level of early implanon discontinuation and factors associated with early discontinuation within 1 year’s duration. In this study, a cross-sectional design and interviewer administered questionnaire was employed among 430 women’s of ever used implanon in Mettu district woreda Ilu Aba Bor Zone. The finding of this study revealed that, Women who did not counseled on the presence of alternatives methods, women who experienced dizziness after insertion of implanon and being having menstrual disturbance after insertion of implanon were significantly associated with early implanon discontinuation. Women who were counseled about the advantage of implanon were protective from early implanon discontinuation. In conclusion, the finding of this study reported a high magnitude of early implanon discontinuation as compared to other previous studies.

## Background

Contraception is an essential public health tool which helps women to avoid unplanned and/or unwanted pregnancies, and prevent unsafe abortions. Contraceptive use helps women space the births of their children, which benefits the health of the mother and child [1].

Implanon is a LARC and extremely effective at preventing pregnancy with a clinical failure rate of less than 1% [2]. It consists of a single thin rod which is 4 cm in length and 2 mm in diameter. Its main mechanism of action is ovulation suppression, augmented by increased cervical mucus viscosity that hinders the passage of spermatozoa and alters the endometrial lining [3]. Despite these, the rate of implanon discontinuation before its due date is not an uncommon problem in various societies because of different associated factors and exposes women to unwanted conception and its consequences [4]. The early or premature discontinuation of implanon can lead to increased rate of unintended pregnancy which is one of the most critical challenges facing the public health system that imposes substantial financial and social costs on society. Besides, when a woman becomes unintentionally pregnant, it can compel her to have unsafe abortion that in turn has a range of complications that can even cost her life.

Early discontinuation of implanon is switching or stopping the method within 12 months of insertion and implanon discontinuation is defined as discontinuation at less than 2.5 years after insertion of implanon [1, 5].

Worldwide, a large number of women become exposed to the risk of conception after contraceptive discontinuation [6]. In sub-Saharan Africa including Ethiopia, there are high population and reproductive health challenges, which are indicating higher maternal morbidity and mortality, higher total fertility rate, unsafe abortion from unintended pregnancies and increasing population growth [4, 7]. In the low income countries implies 16% of women are considered at risk of an unintentional or too closely spaced birth after contraceptive discontinuation [8] and in Ethiopia 42% women were at risk of conception [6].

Since 2009, the Federal Ministry of Health (FMoH) made a strategic decision to launch an implant scale-up initiative and decided to make implanon available at the community level as it is easy to administer by giving trainings for health extension worker (HEWs) on implanon and other health workers (HWs) oriented to the new implanon NXT. Though the effort put by FMOH to scale up implanon, only 8% of currently married women are using it [1].

Besides, when initiating women to use implanon; a poor effort is made to help them continue till the due date. There is limited numbers of variable to dig out factors that are associated with implanon discontinuation. Despite this, there is lack of studies in this issue on this specific study area.

## Methodology

### Study setting and design

A community based cross sectional study design was conducted from October 11 to December 4 2020 G.C at Mettu district, Ilu Abba Bor Zone, Oromia region, south west Ethiopia. Mettu Woreda district is one of the 14 woredas of Ilu Aba Bor zone, located at 620 km to southwest of Addis Ababa. There are 29 kebeles in the woreda with a total population of 85,739 and 18,974 are women in reproductive age group (15–49). There are 34 functional health facilities, 5 health centers and 29 health posts. In all the health centers and health posts, relatively similar composition of health professionals with the same level of health service provision. Moreover, the 29 health posts have at least two health extension workers who provide the same standard of health care services including implanon insertion. Implanon contraceptive is the most commonly used method, especially after safe abortion service. According to woreda health management of information system annual report, about 1054 of women were use implanon contraceptive in the last 1 year.

#### Source population

All women of reproductive age group (15–49) who ever used implanon in Mettu district.

#### Study population

All randomly selected women for whom implanon was inserted over 1-year period, starting from July 8, 2019 to July 7, 2020 GC in Mettu district.

#### Exclusion criteria

Women who are unable to respond due to illness during data collection period was excluded from study.

### Sample size determination and sampling procedure

The sample size was determined by using single population proportion formula for objective one and double population formula for objective two, lastly sample calculated for objective one was becomes higher number and taken for this study. The sample was calculated by considering the proportion of early implanon discontinuation rate of 23.4% from previous study conducted in dale district south nation and nationality of Ethiopia [9], 95% level of confidence, 5% margin of error and 10% non-response rate. Finally, the calculated sample size for objective one was **453**.

### Sampling procedure

Of 29 kebeles found in Mettu woreda district 9 kebeles were selected by using Simple Random Sampling (SRS) method. The number of women whoever used implanon in the last 1 year in the selected kebeles was 841. The sample was proportionally allocated to the selected 9 kebeles. Then, to select the study unit systematic random sampling technique was used. The total number of women used implanon in the last 1 year in the selected HP were 841 and the skip interval was calculated as (K = 841/453 ≈ 2), the first client to be interviewed was selected randomly by lottery method.

### Study variables

#### Dependent variables

Early implanon discontinuation.

Independent variables

#### Sociodemographic characteristics

Age, marital status, women education, religion, woman occupation, partner occupation and partner education.

#### Obstetric factors

Having children during insertion, parity, desire for pregnancy, experience of unplanned pregnancy and previous abortion.

#### Method related factors

Having information about implanon, source of information, counseling, past contraceptive utilization, side effects, appointment for follow up, service satisfaction, reason for choosing implanon, menstrual disturbance and decision maker to use implanon.

### Data collection process

Data collection tool was developed from various similar studies in different parts of the world and modified according to the local context [10–14]. The questionnaire contains three parts: socio-demographic characteristics, obstetric factors and method related factors. Data were collected using interviewer administered structured questionnaire. Data collectors was nine health extension worker staff working in the health post of Mettu woreda district and supervisors was two midwives working in health centers of Mettu woreda district.

### Operational definition

#### Early implanon discontinuation

Removal of implanon by health professionals within 12 months after insertion of the implanon due to different reason.

#### Unintended pregnancy

Pregnancy that occur without the plan of a mother or a father or both.

#### Menstrual disturbance

Menstruation deviant from the normal menses in amount, frequency or duration.

### Data quality management

Pretest was conducted before actual data collection. The questionnaire was translated from English to local language, “Afan Oromo” then, back translated to English to check for consistency by language expert. One-day intensive training was given to the data collectors and supervisors by the principal investigator on the objective of the study, the methods of data collection and how to recruit study participants. Supervisors was observing and supervising data collectors at a time of data collection. At the end of each data collection day, the questionnaire was reviewed and cross checked for completeness, accuracy and consistency by the investigator and corrective discussion was undertaken with all the data collectors. Data was cleaned and edited after it is entered in to the software.

### Data processing and analysis

Data completeness was checked manually, then entered into Epi-data software version 3.1 and exported to SPSS Version 25 for analysis. Descriptive analysis such as frequencies, percent and charts was used to describe the data. Bivariate logistic regression analysis was performed to assess the association of each independent variable and dependent variable. Candidate variables with p-value less than 0.25 were entered to multivariable logistic regressions to identify their association with early implanon discontinuation. The Hosmer and Lemeshow goodness-of-fit test with large p-value (p > 0.05) was checked to see good fitness. Statistical significance was declared at p-value less than 0.05 and 95% CI as cut of point. Odds ratio and 95% CI was used to identify the presence and strength of association.

## Result

### Socio-demographic characteristics of the study participants

In this study, 430 participants have responded to the questionnaires with response rate of 94.9%. The age of study participants was between 17 and 46 years with the mean age of 25.7 ± 5.1 years. Most of the participants (391) 90.9% were married. From the participants, 49.8% of the participants have joined the elementary school while 26.7 and 23.5% of the participants were not educated and have joined secondary school respectively. Whereas 45.6% of them were Muslim by religion, more than have 287 (66.7%) of the women were housewives (Table 1).

**Table 1:** Socio demographic status of women who ever used implanon in Mettu district southwest Ethiopia, 2020 (n = 430)

Variables	Frequency	Percentage (%)
Age		
< 20	72	16.7
21–25	178	41.4
26–30	145	33.7
31–35	19	4.4
> 36	16	3.7
Marital status		
Single	23	5.3
Married	391	90.9
Widowed	5	1.2
Divorced	11	2.6
Woman educational status		
No formal education	115	26.7
Elementary school	214	49.8
Secondary and above	101	23.5
Woman occupation		
House wife	287	66.7
Others (student, employed, merchant)	143	33.3

### Obstetric and gynecologic history of the study participants

Among 430 participants, 386 (89.8%) had live children with a mean year of 2.08 ± 1.151, 348 (80.9%) of women have plan to have children in the future. While women who have experience of unplanned pregnancy were 103 (24%) from the participants 41 (9.5%) have history of abortion at least once (Table 2).

**Table 2:** Obstetric and gynecologic characteristics of women who ever used implanon in Mettu district southwest Ethiopia 2020 (n = 430)

Variables	Frequency	Percentage %
Having children during insertion		
Yes	386	89.8
No	44	10.2
Parity (n = 386)		
1	149	38.6
2–4	216	56.0
5 or more	21	5.4
Plan to have children in the future		
Yes	348	80.9
No	82	19.1
Experience of unplanned pregnancy		
Yes	103	24%
No	327	76
History of abortion		
Yes	41	9.5
No	389	90.5

### Contraceptive related information of the study participants

From 430 participants; 365 (84.9%) of them heard about implanon from different sources such as; friends, health extension workers, midwifes and media. Duration of action is the most heard information among those heard about implanon before insertion 333 (91.2%) (Table 3).

**Table 3:** Contraceptive related information of women who ever used implanon in 2019/2020 in Mettu district southwest Ethiopia 2020 (n = 430)

Variables	Frequency	Percentage %
Ever heard about implanon		
Yes	365	84.9
No	65	15.1
Shifting methods		
Yes	79	18.4
No	351	81.6
Any side effect after insertion		
Yes	347	80.7
No	83	19.3
Counseling before insertion		
Yes	372	86.5
No	58	13.5
Menstrual cycle after insertion		
Regular	32	7.4
Irregular	304	70.7
It already stopped	94	21.9
Use of contraception before		
Yes	295	68.6
No	135	31.4
Decision maker for utilization		
Partner	185	27.1
I my self	388	56.8
Provider	110	16.1
Appointment after insertion		
Yes	308	71.6
No	122	28.4

### Early implanon discontinuation level among women who ever used implanon in Mettu district, southwest Ethiopia, 2020

**Fig. 1:**
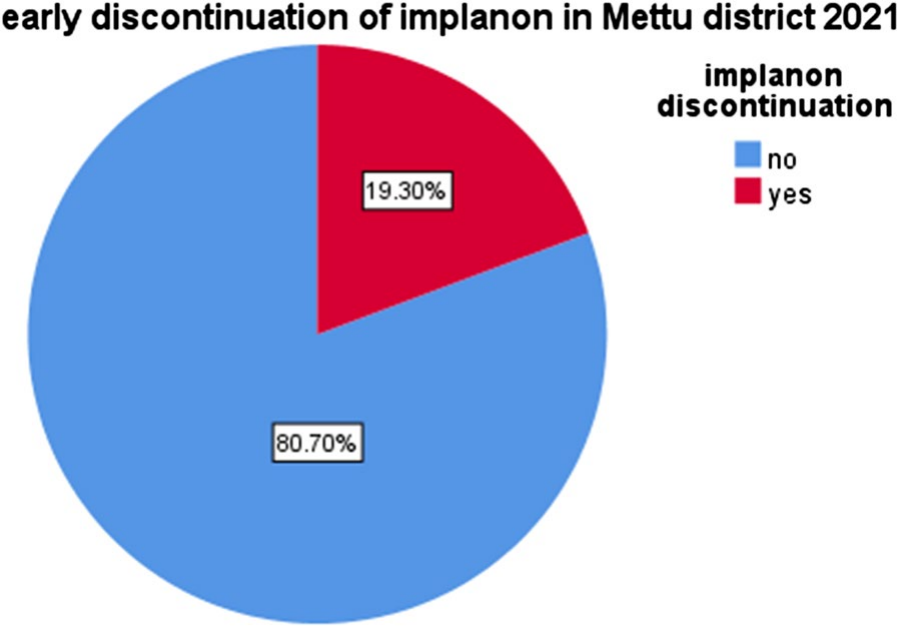
Magnitude of early discontinuation of implanon in Mettu Woreda district

The prevalence of early implanon discontinuation was found to be 89 (19.3%) at 95% CI (15.3–23.0) among women of ever used implanon (Fig. 1).

### Factors associated with early implanon discontinuation among women who ever used implanon in the last 1 year in Mettu district, southwest Ethiopia, 2020

This study revealed that, women who were counseled about the advantage of implanon were also less likely to discontinue early compared to those who did not counseled [AOR: 95% CI 0.49 (0.28–0.87)]. On the other way women who did not counseled about the presence of alternatives were 2.28 times more likely to discontinue when compared to those who did not counseled [AOR: 95% CI 2.28 (1.22–4.26)]. In addition to this; women who experienced dizziness after insertion of implanon were 1.9 times more likely to dis continue implanon early when compared to their counterparts [AOR: 95% CI 1.90 (1.06–3.43)]. The odds of participants who had menstrual disturbance after insertion of implanon were 2.17 times higher than those who hadn’t [AOR: 95% CI 2.17 (1.16–4.08)] (Table 4).

**Table 4:** Factors associated with early implanon discontinuation among women who ever used implanon in 2019/2020 in Mettu district southwest Ethiopia 2020 (n = 430)

Variables	Early implanon discontinuation		COR 95%CI	AOR 95% CI	p-value
	Yes (%)	No (%)			
Having children during insertion					
Yes	81 (21)	305 (79)	1	1	0.052
No	2 (4.5)	42 (95.5)	0.17 (0.04–0.75)	0.23 (0.05–1.01)	
Counseled about advantages					
Yes	22 (13.5)	141 (86.5)	1	1	0.015*
No	61 (22.8)	206 (77.2)	0.52 (0.30–0.89)	0.49 (0.28–0.87)	
Counseled about presence of alternatives					
Yes	16 (10.7)	133 (89.3)	1	1	0.009*
No	67 (23.8)	214 (76.2)	2.60 (1.44–4.68)	2.28 (1.22–4.26)	
Decide by them selves					
No	72 (18.6)	316 (81.4)	1	1	0.066
Yes	11 (26.2)	31 (73.8)	1.55 (0.74–3.24)	2.15 (0.94–4.90)	
Experience of unplanned pregnancy					
Yes	12 (11.7)	91 (88.3)	0.47 (0.24–0.91)	0.59 (0.29–1.19)	0.147
No	71 (21.7)	256 (78.3)	1	1	
Dizziness					
Yes	27 (30.7)	61 (69.3)	2.26 (1.32–3.86)	1.90 (1.06–3.43)	0.031*
No	56 (16.4)	286 (83.6)	1	1	
Menstrual disturbance after insertion					
Yes	67 (23.4)	219 (76.6)	2.44 (1.36–4.40)	2.17 (1.16–4.08)	0.015*
No	16 (11.1)	128 (88.9)	1	1	
Headache					
Yes	43 (23.5)	140 (76.5)	1.58 (0.98–2.57)	1.57 (0.92–2.67)	0.096
No	40 (16.2)	207 (83.3)	1	1	

*COR* Crude odd ratio, *AOR* adjusted odds ratio

*p-value < 0.05

## Discussion

The finding of this study revealed that early discontinuation rate of implanon in Mettu district was 19.3%. This goes in line with study conducted in Hawassa which is 16% [15]. On the other hand, this study has shown that early discontinuation of implanon is higher than studies in Upper Egypt [16] and Ethiopian Demographic and Health Survey which is [1].

Conversely the findings were found to be lower than a study done in South Africa where about (67.3%) of the women discontinued contraceptive implanon early [10]. This deviation might be due to sample size, study design, difference in the study setting and socio-cultural and socio demographic variability.

With regard to counseling; previous studies that were done in different areas of our country discuss the association of counseling in general with early implanon discontinuation and failed to mention specific components [9, 17–19]. But in this study it was found out that, counseling about advantages of implanon was one of the factors that had significant association with early implanon discontinuation. Women counseled about the advantage of implanon were 51% times less likely to discontinue early compared to those who were not counseled. This can be explained by the fact that sufficient understanding about the advantages of implanon helps the women to retain it for longer period. This result is almost similar with study conducted in Hawassa [15]. On the other hand, those women who were not counseled about presence of alternative contraceptive methods were 2.28 times more likely to discontinue implanon early. This is because those women who were adequately counseled about the pros and cons of all available contraceptive methods will have the opportunity to understand the benefit of retaining the implanon than removing it earlier than the due date.

Women who experienced dizziness after insertion of implanon were 1.9 times more likely to discontinue implanon early when compared to their counterparts. This might be due to its interference with their daily activities and unsatisfactory management. Even if most studies discussed about the overall counseling service given; this result is almost similar with different studies [5, 9, 17, 19].

The odds of participants who had menstrual disturbance after insertion of implanon were 2.17 times more likely to discontinue implanon early compared to those who hadn’t. This could be due to poor counseling on the possible side effects like menstrual disturbance and poor management of the occurred menstrual disturbance. Besides, the presence of menstrual irregularity might affect their daily activities and the intimacy with their spouses.

This finding is found to be consistent with study in Kinshasa as this study implies those women who developed heavy menstrual bleeding had a positive association with early discontinuation of implanon when compared to their counter parts [20]. Similarly study in Hawassa revealed that women who suffered from abnormal bleeding were more likely to discontinue implanon early when compared to their other part [15]. Correspondingly Australian women who were not experiencing irregular menstrual cycle after insertion of implanon were less likely to discontinue implanon early when compared to their corresponding women [21]. The current study lacks qualitative and comprehensive study including health professionals and giving emphasis on quality of effective counseling is recommended for further researcher.

## Conclusion and recommendation

Implanon discontinuation rate in this study was high and factors associated with early discontinuation were counseling about advantages of implanon, counseling about presence of alternative methods, experiencing menstrual disturbance and dizziness after insertion of implanon. Hence, to avert early implanon discontinuation health care providers should be given proper pre insertion counseling with giving emphasis on the advantages and possible side effects.

## Data Availability

Not applicable.

## Abbreviations

AOR: Adjusted odds ratio
COR: Crude odds ratio
FP: Family planning
GC: Gregorian calendar
FMoH: Federal Ministry of Health
HEW: Health extension worker
HW: Health workers
HP: Health post
LARC: Long acting reversible contraceptive
SPSS: Statistical Package for Social Science

## References

[1] Zerfu TA. Ethiopia Demographic and Health survey. 2017;(September):158. https://www.researchgate.net/publication/320109833

[2] FPA. Your guide to long-acting reversible contraception (LARC). Public Health Engl. 2017;(september):1–2.

[3] WHO/RHR and Johns Hopkins Bloomberg School of PH/CCP. Family planning. Global handbook for providers updated 3rd edition. Knowledge for health project. Family planning: a global handbook for providers (2018 update). WHO; 2018. 1–460 p.

[4] WHO. Contraception fact sheet. Hum Reprod Programe. 2014;4. http://apps.who.int/iris/bitstream/10665/112319/1/WHO_RHR_14.07_eng.pdf?ua=1.

[5] G/Medhin T, Gebrekidan KG, Nerea MK (2019). Early implanon discontinuation rate and its associated factors in health institutions of Mekelle City, Tigray, Ethiopia 2016/17. BMC Res Notes.

[6] Ali MM, Cleland JG, Shah IH, WHO (2012). Causes and consequences of contraceptive discontinuation: evidence from 60 Demographic and Health Surveys.

[7] Yalew SA, Zeleke BM, Teferra AS (2015). Demand for long acting contraceptive methods and associated factors among family planning service users, Northwest Ethiopia: a health facility based cross sectional study. BMC Res Notes.

[8] Staveteig S, Mallick L, Winter R. Discontinuation of long-acting reversible contraceptives (LARCs) in low-income countries: the role of method access and programmatic quality.

[9] Nageso A, Gebretsadik A (2018). Discontinuation rate of Implanon and its associated factors among women who ever used Implanon in Dale District Southern Ethiopia. BMC Womens Health.

[10] Seekoe E, Ajayi AI (2018). Reasons for discontinuation of implanon among users in Buffalo City Metropolitan Municipality, South Africa: a cross-sectional study. Afr J Reprod Health.

[11] Abebe SM. Explaining LARCs discontinuity in Ethiopia: the experience of women who access contraceptives in selected public health facilities. 2017;1–19.

[12] Gelaw YM, Sciences H, Asresie MB, Sciences H, Anteneh ZA, Sciences H. Level and timing of implanon discontinuation and associated factors among women who used implanon in Andabet District, public health facilities. 2017;1:1–15. 10.1155/2021/6647660 PMC8363448 34395623

[13] Asaye MM, Nigussie TS, Ambaw WM. Early implanon discontinuation and associated factors among implanon user women in Debre Tabor Town, Public health facilities, Northwest Ethiopia, 2016. 2018;2018. 10.1155/2018/3597487 PMC5896376 29796392

[14] Utaile MM, Debere MK, Nida ET, Boneya DJ (2020). A qualitative study on reasons for early removal of Implanon among users in Arba Minch town, Gamo Goffa zone, South Ethiopia: a phenomenological approach. BMC Womens Health.

[15] Tegegne K (2019). Early discontinuation of implant and its associated factors among women who ever used implant in 2017/2018 in Hawassa. J Med Case Rep.

[16] Aziz M, El-Gazzar A, Elgibaly O (2018). Factors associated with first-year discontinuation of Implanon in Upper Egypt: clients’ and providers’ perspectives. BMJ Sex Reprod Health.

[17] Mamo K, Siyoum M. Premature implanon discontinuation and associated factors among implanon user women in Ambo town, Central Ethiopia. J Health Med Nurs. 2019;58.

[18] Amanuel T. Determinants of early implanon discontinuation among women who ever used implanon in Duguna Fango woreda, Wolayta zone, southern Ethiopia. PMC. 2017;(June).

[19] Siyoum M, Mulaw Z, Abuhay M, Kebebe H (2017). Implanon discontinuation rate and associated factors among women who ever used implanon in the last three years in Debre Markos Town, Northwest Ethiopia. ARC J Public Health Community Med.

[20] Akilimali PZ, Hernandez J, Anglewicz P, Kayembe KP, Bertrand J (2020). Incidence and determinants of Implanon discontinuation: findings from a prospective cohort study in three health zones in Kinshasa, DRC. PLoS ONE.

[21] Weisberg E, Bateson D, Mohapatra L, Understandings C (2014). A three-year comparative study of continuation rates, bleeding patterns and satisfaction in Australian women using a subdermal contraceptive implant or progestogen releasing-intrauterine system. Eur J Contracept Reprod Health Care.

